# Adiponectin and Intelectin-1: Important Adipokine Players in Obesity-Related Colorectal Carcinogenesis

**DOI:** 10.3390/ijms18040866

**Published:** 2017-04-19

**Authors:** Keisuke Kawashima, Kenichi Maeda, Chiemi Saigo, Yusuke Kito, Kazuhiro Yoshida, Tamotsu Takeuchi

**Affiliations:** 1 Department of Pathology and Translational Research, Gifu University Graduate School of Medicine, Yanagido, Gifu 501-1193, Japan; kaw2path@gifu-u.ac.jp (K.K.); chiemi3150@yahoo.co.jp (C.S.); kitoysk@gifu-u.ac.jp (Y.K.); 2 Department of Surgical Oncology, Gifu University Graduate School of Medicine, Yanagido, Gifu 501-1193, Japan; maeken77@hotmail.com (K.M.); kyoshida@gifu-u.ac.jp (K.Y.)

**Keywords:** adipokines, obesity, colorectal cancer, adiponectin, intelectin-1, TMEM207

## Abstract

Overweight is believed to be associated with colorectal cancer risk. Adipose tissue is loose connective tissue composed of adipocytes. It is now recognized as a major endocrine organ, secreting humoral factors collectively called adipokines. Aberrant hormonal systems consisting of modulated adipokines and their receptors are thought to play a role in colorectal carcinogenesis and cancer progression in obese conditions. However, it is still unclear whether and how each adipokine relates to colorectal carcinogenesis. Notably, a couple of molecules that were initially proposed to be obesity-related adipokines were disqualified by subsequent studies. The adipokines, adiponectin, and intelectin-1 (also known as omentin-1), whose levels are decreased in obesity, act as tumor suppressor factors in various cancers. Numerous studies have demonstrated a link between the insufficient expression and function of adiponectin and its receptor, T-cadherin, in colorectal carcinogenesis. Moreover, our recent study indicated that loss of TMEM207, which is critical for the proper processing of intelectin-1 in the colon mucosa, leads to insufficient intelectin-1 production, thus participating in colorectal carcinogenesis. Here, we discuss the recent understanding of the role of adipokines in colorectal carcinogenesis and subsequently describe the potent tumor suppressor roles of intelectin-1 and TMEM207 in colorectal cancer.

## 1. Overweight/Obesity and Colorectal Cancer: Epidemiological Studies

Various epidemiological studies have demonstrated that overweight/obesity is a contributing factor to higher incidence and mortality of colorectal cancer [1,2]. Data from HPFS (US Health Professionals Follow-Up Study) indicate that overweight is a modifiable risk factor for colon cancer among men [3]. A Canadian case-control study suggested that obesity is associated with increased risk of sporadic and Lynch syndrome-related colorectal cancer in men [4] (Lynch syndrome is a hereditary non-polyposis form of colorectal cancer [5]). Norwegian health surveys reported that obesity in childhood and adolescence doubles the risk of death from colon cancer regardless of gender [6]. These meta-epidemiological analyses consistently highlight the association between overweight/obesity and colorectal cancer risk, especially in men.

## 2. Adipokine-Receptor Axis in Colorectal Carcinogenesis

**Figure 1:**
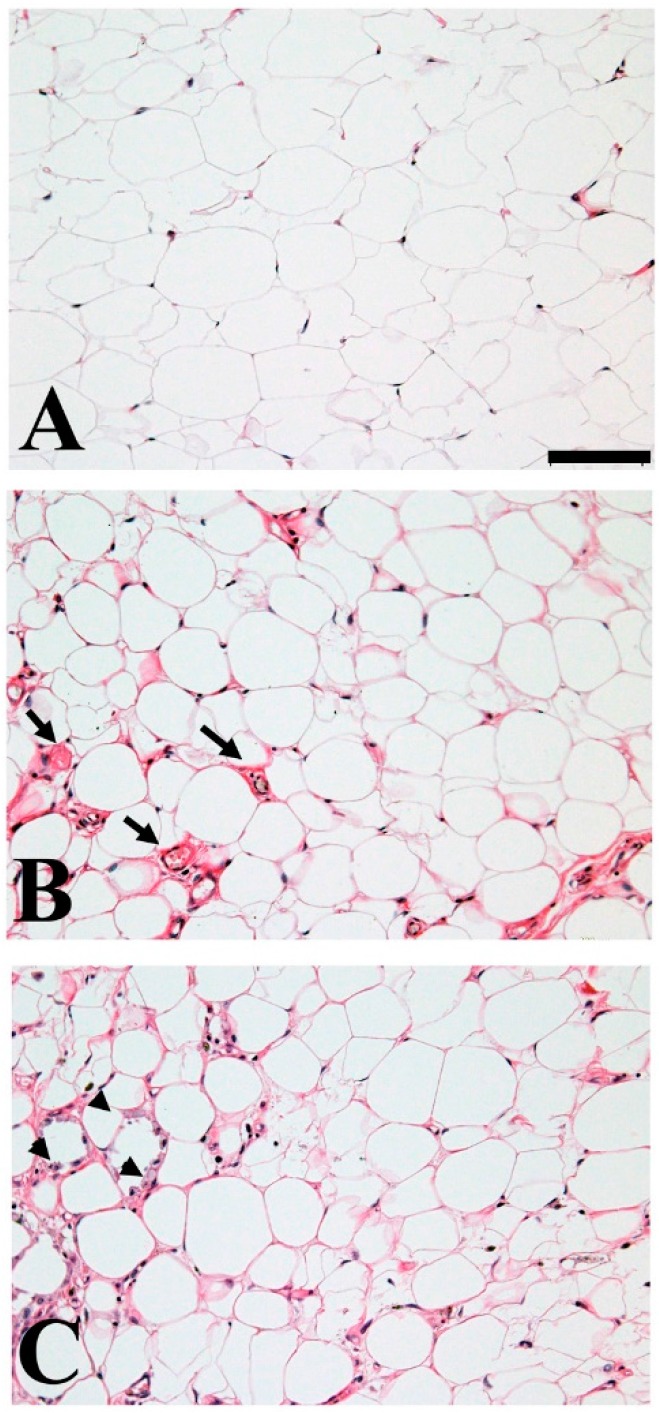
Morphological alternation of human adipose tissue by inflammation. Adipose tissue dynamically changes by excess nutrition, overweight/obese. During the process of hypertrophy, heathy adipose tissue (**A**) may increase vascularity (**B**) arrow indicates the vascular vessels, furthermore, may harbor inflammation (**C**) arrow head indicates the macrophage infiltration. Adiponectin and intelectin-1 secretion from adipocyte or adipose stromal cells are decreased by hypertrophy, instead pro-inflammatory adipokines—i.e., Leptin secretion—is upregulated. Formalin-fixed, paraffin-embedded archival pathological tissue sections were stained with hematoxylin and eosin after obtaining approval from the Institutional Review Board of the Gifu University Graduate School of Medicine (Specific approval No. 24-256). The scale bar represents 100 µm.

Adipocytes are critical for maintaining biological homeostasis as they constitute storage depots for triglycerides and secrete humoral factors [7]. Adipocyte hypertrophy is accompanied by increased vascularity and inflammation (Figure 1). Moreover, these alterations can increase or decrease the expression of adipose stromal cell-related adipokines. Recent studies revealed that the loss or overexpression of several adipokines and the aberrant expression of their receptors may lead to colorectal carcinogenesis (Table 1).

### 2.1. Adiponectin in Colorectal Carcinogenesis

Adiponectin is the most abundant circulating peptide hormone, accounting for approximately 0.01% of total plasma protein [8,9,10,11]. It is found in serum as three distinct oligomers, namely a trimeric, a hexameric, and a high-molecular-weight (HMW, composed of 12–18 adiponectin molecules) form [12,13]. Most investigators now believe HMW adiponectin to possess higher biological activity compared to trimeric or hexameric adiponectin [14,15,16]. The levels of total plasma adiponectin as well as those of the HMW fraction are strongly positively correlated with insulin sensitivity [17]. Paradoxically, even though adiponectin is exclusively secreted by adipocytes, the serum concentration of total adiponectin is reduced in obesity [11].

Wei et al. reported that low plasma adiponectin levels were associated with the risk of colorectal cancer in men [18]. Moreover, Otake et al. showed that a low adiponectin level was a stronger risk factor than a high triglyceride level or body mass index in patients with adenoma and early colorectal cancer [19]. Interestingly, adiponectin inhibits the growth of cultured colorectal cancer cells in vitro via activation of adenosine monophosphate-activated protein kinase (AMPK) and suppresses the mammalian target of rapamycin (mTOR) pathway [20,21].

The canonical adiponectin receptors, AdipoR1 and AdipoR2, are expressed in both normal colon epithelium and colorectal cancer [22]. By contrast, there are many reports which indicate insufficient T-cadherin (also known as CDH13 and H-cadherin) expression, the third receptor of adiponectin [23], in colorectal carcinogenesis. T-cadherin protein is a receptor of the hexameric and HMW isoforms of adiponectin [24]. First, Toyooka et al. showed that hypermethylation of the promoter region of the *T-cadherin* gene is frequently found in colorectal cancers and adenomas [25]. Subsequently, Hibi et al. found that found that almost all (83%) poorly differentiated colorectal cancers presented *T-cadherin* methylation [26]. Interestingly, Scarpa et al. recently reported the methylation status of the T-cadherin promoter in non-neoplastic mucosa as a marker of ulcerative colitis-associated colorectal cancer [27]. The downregulation of T-cadherin expression may be linked to the deficient function of adiponectin in colonic mucosa.

In conclusion, the insufficient expression and/or function of the adiponectin-T-cadherin axis may lead to colorectal carcinogenesis.

### 2.2. Leptin in Colorectal Carcinogenesis

Leptin (from the Greek word “leptos”, meaning “thin”) is a 16 kDa protein hormone. It was the first adipokine to be identified [28,29]. Leptin receptors belong to the class I cytokine receptor family, which consists of single-membrane spanning receptors hallmarked by the presence of one or more cytokine receptor homology domains possessing the WSXWS motif in the extracellular portion adjacent to the cell membrane [30]. Although six isoforms of the leptin receptor have been identified, only two have thus far been linked to intracellular signaling, of which only the longest isoform (OBRb) has full signaling capability [31]. OBRb is highly expressed in areas of the hypothalamus and controls food intake to achieve energy balance and regulate body weight. In obesity, sensitivity to leptin decreases, resulting in the inability to detect satiety despite the abundance of stored energy. 

There are contradictory reports on the pathobiological properties of leptin in the onset and progression of colorectal cancer. In several studies, a significant increase in colon cancer risk with higher serum leptin concentration was observed [32], while others reported significantly lower leptin levels in cancer patients compared to controls [33,34,35]. It has also been reported that the leptin expression level is inversely associated with the nodal stage [36]. To sum up, the associations between leptin levels and the risk of colorectal cancer or adenoma are still unclear.

Molecular biology experiments in vitro and animal model experiments also failed to reach a conclusion. Aparicio et al. reported that leptin significantly stimulated DNA synthesis in colon cancer cells, but did not promote the growth of colon cancer cells in a xenograft assay [37]. Moreover, they demonstrated that hyperleptinemia in *Apc^Min/+^* mice, which harbor a point mutation at the *Apc* gene and are a well-established model for human familial adenomatous polyposis [38], did not enhance the development of intestinal adenomas. Higurashi et al. generated intestinal epithelium-specific OBRb conditional knockout mice and concluded that OBRb-mediated signaling is important for the progression of aberrant crypt foci to colonic tumors [39].

Two critical points must be further examined to determine whether the leptin-OBRb axis plays a role in colorectal carcinogenesis. First, the detailed expression status of OBRb should be evaluated in human colorectal cancer tissue specimens. OBRb is expressed in human colon cancer cell lines and adenoma tissue specimens [40]. A previous report detected OBRb immunoreactivity in colorectal adenocarcinoma tissue specimens using a conventional goat antibody, C-20 (Santa Cruz Biotechnology, Santa Cruz, CA, USA), which is no longer available and has been substituted with a specific monoclonal antibody to OBRb (sc-8391, Santa Cruz Biotechnology) [41]. Re-evaluation of OBRb expression in colorectal cancer cells in vivo, particularly the relationship between expression and prognosis, is necessary. Second, the pathobiological properties of the soluble form of OBRb should be evaluated in colorectal carcinogenesis. The soluble leptin receptor binds leptin and modulates steady-state leptin levels by complexing free leptin in the circulation [42]. Aleksandrova et al. reported a strong inverse association between circulating soluble OBRb and colorectal cancer risk, which was independent of leptin concentrations [43]. Further research is needed to determine the relationship of soluble leptin receptor and leptin in colorectal carcinogenesis.

In summary, whether the leptin-OBRb axis plays a role in colorectal carcinogenesis remains unclear.

### 2.3. Is Resistin Important for Colorectal Carcinogenesis?

Resistin is a 12.5 kDa cysteine-rich peptide that induces low-grade inflammation by stimulating monocytes in humans. It owes its name to its ability to abrogate insulin function (resulting in resistance to insulin) [44]. Accordingly, previous studies reported that the serum resistin level was significantly higher in patients with colorectal cancer than in healthy controls [35,45]. Overexpression of a resistin receptor, adenylyl cyclase-associated protein 1 (CAP1) [46], was also reported in epithelial ovarian cancer [47] and breast cancer [48], which are also known as obesity-related malignant tumors.

However, recent reassessments have shown that resistin seems to be of greater relevance in relation to the immune stress response than in the regulation of glucose homeostasis [49]. Surprisingly, human studies failed to link resistin to insulin resistance [50]. Under these circumstances, CAP1 expression has not been clarified in colorectal cancer 

The pathobiological role of the resistin-CAP1 axis in obesity-related colorectal carcinogenesis remains largely unknown. Notably, Huang et al. reported that treatment of both HCT-116 and SW-48 colon cancer cells with resistin increased the adhesion of both cells to human umbilical vein endothelial cells [51]. Further experimental studies may reveal the role of resistin in colorectal carcinogenesis.

### 2.4. Is Visfatin a True Adipokine with Possible Relation to Colorectal Cancer?

Visfatin is a highly-conserved 52 kDa protein found in living species from bacteria to humans. It was initially reported to be expressed in the visceral adipose tissue, with its expression correlating with the body mass index and commonly increasing in metabolic disease [52], this finding was later retracted.

Yang et al. reported that visfatin can trigger the epithelia-mesenchymal transition of colorectal cancer cells through Akt/GSK-3β/β-catenin signaling and suggested that increased expression of visfatin results in more aggressive colorectal cancer [53].

However, the contribution of adipose tissue to circulating visfatin levels was not established. Recent studies, which prefer referring to this protein as nicotinamide phosphoribosyltransferase (NAMPT), characterized it as a cytoplasmic enzyme that regulates intracellular NAD levels—the cellular redox state—and histone deacetylases, in addition to promoting cell proliferation and inhibiting apoptosis [54]. In the end, the original report that “identified visfatin as a new protein found in adipose tissue that has insulin-mimetic properties” was retracted.

The pathobiological properties of visfatin in obesity-related colorectal carcinogenesis remain unclear. Examining the role of resistin in obesity-related colorectal carcinogenesis remains difficult.

### 2.5. Apelin, the Ligand of the Seven-Transmembrane G Protein-Coupled Receptor APJ, in Colorectal Cancer 

Initially, apelin was identified as the ligand of an “orphan” G protein-coupled receptor, designated the APJ receptor (a putative receptor protein that is related to the *angiotensin*-type 1 receptor but does not bind angiotensin II) [55]. Apelin is composed of 77 amino acids and has a signal peptide. The mature, biologically active form of apelin is known as apelin 13 (aa 65–77). Apelin is ubiquitously expressed by cells in various tissues, including adipocytes.

Apelin is upregulated in the adipose tissues of obese subjects; thus, plasma apelin concentration is increased in obesity and metabolic disease [56]. Picault et al. showed that both apelin and the APJ receptor were overexpressed in colorectal cancer and promoted cancer progression in an autocrine manner [57]. They also demonstrated that apelin protected LoVo colon cancer cells from apoptosis by inactivating a caspase-dependent pathway and decreasing the degradation of poly (ADP-ribose) polymerase. Furthermore, they showed that treatment with an APJ antagonist reduced LoVo proliferation in vitro. The results of Sorli et al. also supported a role for apelin in colorectal cancer [58]. Specifically, they detected apelin gene upregulation in 7 of 20 colorectal cancers using cancer-profiling arrays on cDNA samples from normal and tumor tissues of the same patient. They also reported that apelin is a potent activator of tumor neoangiogenesis. Finally, Kidoya et al. showed that the apelin/APJ system induced maturation of tumor vasculature [59].

These findings imply that antagonists of the apelin/APJ system can prevent the progression of colorectal cancer by impairing tumor cell growth and/or inhibiting tumor neovascularization.

## 3. Intelectin-1 in Colorectal Carcinogenesis

Human intelectin-1 (“intestinal lectin”, also known as ITLN-1 and Omentin-1) is a 34-kDa secretory protein that was first identified by its ability to bind galactofuranose units in the carbohydrate chains of bacterial cell walls [60]. It was thus recognized as a player in innate immunity against bacteria. Subsequently, intelectin-1 was reported to be highly expressed in the visceral adipose tissue, especially in stromal vascular cells and to a lesser extent in adipocytes [61]. In vitro studies have shown that intelectin-1 increases insulin signal transduction by activating protein kinase B (also known as Akt) and enhances insulin-stimulated glucose transport in isolated human adipocytes. Notably, the plasma level of intelectin-1, as was also the case with adiponectin, decreases in cases of obesity and is associated with insulin resistance [61,62,63].

A series of reports indicated the potential role of intelectin-1 in carcinogenesis [64,65,66,67,68,69,70,71]. With respect to gastrointestinal carcinogenesis, Li et al. reported that intelectin-1 increased the levels of hepatocyte nuclear factor 4 α (HNF4α), resulting in the suppression of the nuclear translocation and transcriptional activity of β-catenin in gastric cancer cells [70]. They also concluded that high intelectin-1 levels were significantly correlated with better outcomes in patients with gastric cancer. In colorectal cancer, Kim et al. identified intelectin-1 as a marker of favorable outcome in stage IV cancer [66]. These findings indicate that intelectin-1 functions as a tumor suppressor in gastrointestinal cancers. In contrast, Aleksandrova et al. reported that a higher intelectin-1 concentration was associated with a higher colorectal cancer risk in a prospective cohort study [72]. These controversial findings may reflect the “*Janus*-faced” pathobiological properties of intelectin-1 in various types of carcinogenesis.

Intelectin-1 was identified as a human intestinal lactoferrin receptor [73]. However, human intelectin-1 has an N-terminal signal peptide without a putative transmembrane domain or potential GPI-modification site. Therefore, intelectin-1 may be a canonical extracellular protein or secretory molecule. An un-identified surface membrane protein may act as a receptor of intelectin-1 in colorectal cancer. Further fundamental biological studies are needed to understand the role of intelectin-1 in colorectal carcinogenesis.

## 4. TMEM207, a Protein Fostering Proper Processing of Intelectin-1, in Colorectal Carcinogenesis

TMEM207 was identified by researchers in a comprehensive project called the Secreted Protein Discovery Initiative, which was aimed at finding novel secretory and transmembrane proteins [74]. Subsequently, TMEM207 is identified as an invasion activity-related molecule in gastric signet-ring cell carcinoma [75]. Interestingly, transgenic mice in which murine TMEM207 is overexpressed in cutaneous hair follicle bulge cells spontaneously develop a cutaneous appendage tumor [76]. 

Recently, we found that a transmembrane protein, designated TMEM207, facilitated the proper processing of intelectin-1 [77]. The siRNA-mediated downregulation of TMEM207 resulted in polyubiquitination followed by proteasome degradation of intelectin-1 and decreased intelectin-1 secretion by colorectal cancer cells. 

TMEM207 expression was detected in 38 of 216 colorectal cancer tissue samples and displayed a significant inverse correlation with lymph node metastatic status. Its expression also significantly correlated with the mucinous phenotype of colorectal carcinoma (*p* = 0.01). Specifically, TMEM207 was expressed in 24 of 34 mucinous colon cancers but only in 10 of 178 non-mucinous cancer tissue specimens.

**Figure 2:**
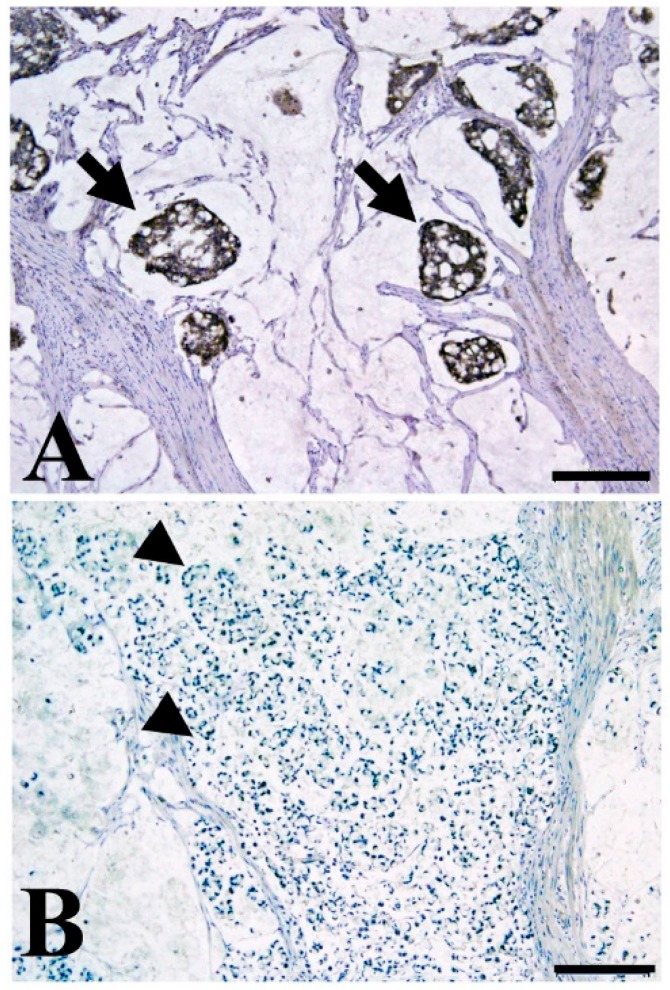
Representative immunohistochemical staining of mucinous colon cancer with TMEM207 immunoreactivity (**A**) and without immunoreactivity (**B**); (**A**) Note the strong immunoreactivity to the specific antibody to TMEM207 in mucinous carcinoma with favorable prognosis (stage III, disease-free survival of over 60 months). Arrow indicate the TMEM207 immunoreactivity in mucinous carcinoma cells; (**B**) Little or no TMEM207 immunoreactivity was found in patients with poor prognosis. Arrow head indicate the negative TMEM207 staining. Archival pathological colorectal cancer tissues including mucinous carcinoma were immunostained with a conventional rabbit antibody to the synthetic peptide VNYNDQHPNGW (amino acid residues 40–50 of TMEM207). Details of the immunohistochemical staining procedure were described previously [77]. Briefly, antigen retrieval in deparaffinized tissue slices was performed with 0.25% trypsin for 5 min at 37 °C. After incubation for 30 min in 10% normal goat serum, the slides were incubated with various antibodies overnight at 4 °C. We used the ImmPRESS Polymerized Reporter Enzyme Staining System (Vector Laboratories Inc., Burlingame, CA, USA). The present study was conducted in accordance with the ethical standards of the Helsinki Declaration in 1975, after obtaining approval from the Institutional Review Board of the Gifu University Graduate School of Medicine (specific approval number 25-81). Immunoreactivity was based on examination of five high-power (×400) microscopic fields or the total tumor (when the tumor was smaller than five fields) for each case. Tumors showing strong immunoreactivity in >5% of cancer cells were considered positive. The scale bar represents 200 µm.

Very recently, we examined the prognostic value of TMEM207 in stage III or IV mucinous colorectal carcinoma. Interestingly, TMEM207 expression was significantly associated with favorable prognosis (*p* = 0.014) (Figure 2). Thus, the intelectin-1/TMEM207 axis might be a prognostic biomarker of colorectal carcinomas, especially in the case of the mucinous type. 

Further examination to unravel the pathobiological property of intelectin-1/TMEM207 axis in mucinous carcinoma of the colorectum is now underway.

## 5. Conclusions and Future Perspectives

We summarized studies of aberrant adipokines/receptors in colorectal carcinogenesis in Table 1. Recent advances indicate that adiponectin may function as a tumor suppressor in colorectal carcinogenesis, while the apelin/APJ system may contribute to colorectal carcinogenesis. Low-level adiponectin is thought to be associated with a higher risk of colorectal cancer. Interestingly, Inamura et al. recently showed that low-level adiponectin is associated with KRAS-mutated colorectal cancer, but not with KRAS wild-type colorectal cancer [78].

Thus, a possible replacement therapy for adiponectin, particularly for KRAS-muted colorectal cancer and the development and application of apelin/APJ antagonists, should be pursued. Further studies are needed to determine whether intelectin-1 acts as a tumor suppressor or promoter in colorectal carcinogenesis.

## Figures and Tables

**Table 1:** Representative carcinogenesis related adipokines and receptors in colorectal cancer.

Aberrancy of Adipokines/Receptors in Colorectal Carcinogenesis		References
**Adiponectin/T-cadherin**	The insufficient expression and/or function of the adiponectin-T-cadherin axis may lead to colorectal carcinogenesis.	[18,19,25,26,27]
	a: Low plasma level is associated with risk for colorectal cancer.	
	b: Loss of T-cadherin expression due to aberrant methylation of *T-cadherin* gene promoter in colorectal cancer.	
**Leptin**	Discrepant observation.	[32,33,34,35]
	a: Significant increase in risk of colon cancer with increasing serum levels of leptin.	
	b: Significantly lower serum leptin levels in colon cancer patients as compared to controls.	
**Resistin**	Putative biomarker of colorectal malignant potential and stage progression.	[35,45,51]
**Visfatin**	Now recognized as a cytoplasmic enzyme “nicotinamide phosphoribosyltransferase”.	[54]
**Apelin**	Overexpression in colorectal cancer cells and tissues.	[57,58]
	a: Apelin protects colon cancer cells from apoptosis.	
	b: Apelin contributes tumor neovascularization.	
**Intelectin-1 (Omentin-1)**	a: Downregulation of intelectin-1 is related to the unfavorable prognosis among patients with colorectal carcinoma at an advanced stage.	[66,77]
	b: Loss of TMEM207, which participates proper processing of intelectin-1, promotes colorectal carcinogenesis.	
